# Supplementary material for: Associative nature of event participation dynamics: A network theory approach
Source: PLoS One. 2017 Feb 6;12(2):e0171565. doi: 10.1371/journal.pone.0171565 (PMC5293197; doi:10.1371/journal.pone.0171565)
Supplement: S1 File — The probability distribution P(x) of total numbers of participations in group events x, obtained from the empirical data for the four selected Meetup groups (blue circles). We also show truncated power law fit x−αe−Bx (solid lines), power law fit x−γ (dotted-dashed lines), and exponential fit e−λx (dotted lines). Fig A Log likelihood ratio R and the π-value compare fits to the power law and fits to the truncated power law for the probability distribution of total numbers of participations in group events. Table A The probability distribution of successive numbers of participations in group events xS, for the four selected Meetup groups. The probability distribution follows power law behavior P(xS)∼xS-γ. Fig B The probability distribution of time lags between two successive participations in group events yS, for the four selected Meetup groups. The probability distribution follows truncated power law behavior P(yS)∼yS-αe-ByS. Fig C The probability distribution of link weights in a weighted network before and after filtering, for the four selected Meetup groups. Fig D The dependence of a degree strength ratio on the number of participations, averaged over all members for the four considered Meetup groups. Red circles correspond to results obtained from empirical data, while blue squares correspond to randomized data. Fig E The dependence of group members’ average degree 〈q〉 and strength 〈s〉 on numbers of participations for a real weighted network and a randomized network. Fig F The dependence of group members’ average non-weighted 〈ci〉 and weighted clustering coefficient 〈ciW〉 on numbers of participations for a real weighted network and a randomized network. Fig G The probability distribution of relative size fluctuations 〈e〉-e〈e〉, for the four considered Meetup groups, where e is the event size and 〈e〉 is the average event size. Fig H. (PDF) [file pone.0171565.s001.pdf]

Supplementary Information: Associative nature of event participation dynamics: a network theory approach

Jelena Smiljanić[1,2 *] and Marija Mitrović Dankulov[1]

[1]Scientific Computing Laboratory, Center for the Study of Complex Systems, Institute of Physics Belgrade, University of Belgrade, Pregrevica 118, 11080 Belgrade, Serbia
[2]School of Electrical Engineering, University of Belgrade, P.O. Box 35-54, 11120 Belgrade, Serbia
[*]jelenas@ipb.ac.rs

December 26, 2016

1 Network filtering

The Meetup dataset, containing information on organised events by certain Meetup group and members of that group that confirmed attendance at an event, allows us to construct member-event bipartite network with adjacency matrix B. For each member $i \in \{1, \ldots, N\}$ and event $l \in \{1, \ldots, M\}$, matrix element $B_{il} = 1$ if member i participated event l, or $B_{il} = 0$, otherwise. The degree of member i is defined as the total number of events member i participated in, $k_i = \sum_l B_{il}$, and similarly, the degree of event l is defined as the total number of members attended the event, $d_l = \sum_i B_{il}$. Given the matrix B, social relations between Meetup members can be analysed using the projected unipartite member-member weighted network, where the weight of the link between two members is equal to the number of events they both attended. The observed weighted network is the dense network where some of the non-zero edges can be a matter of coincidence. For instance, two frequent attendees can meet several times due to a chance not due to the fact that there is some relation between them, which means that the connection between them is not significant for our analysis. Also, the connections between members that meet at big events and never again can not be regarded as social relations and thus they need to be excluded from our analysis. To make the distinction between significant and non-significant edges is nontrivial task [1–3]. Here we use the method which enables us to calculate the significance of the link between two members based on the probability for that link to occur in random network. As a null model we use configuration model of bipartite network [2–5].

First we describe general framework for constructing randomized network ensemble $\mathcal{G}$ with given structural constraints $\{x_i\}$. The maximum-entropy probability of the graph in the ensemble, $P(G)$, is given by

$$P(G) = \frac{1}{Z} e^{-\sum_i \lambda_i x_i}, \tag{1}$$

where the λ_i are Lagrangian multipliers and the partition function of these network ensembles is defined as

$$Z = \sum_G e^{-\sum_i \lambda_i x_i}. \tag{2}$$

The ensemble average of a graph property x_i can be expressed as

$$\langle x_i \rangle = \sum_G x_i(G)P(G) = -\frac{\partial}{\partial \lambda_i} \ln Z. \tag{3}$$

Then the constants λ_i could be determined from (3).

Let us now consider configuration model of the member-event bipartite network with given degree sequence k_i and d_l. In this case the partition function can be written as

$$Z = \sum_G e^{-\sum_i \alpha_i k_i - \sum_l \beta_l d_l} = \sum_G e^{-\sum_{il}(\alpha_i+\beta_l)B_{il}} = \prod_{il}(1 + e^{-(\alpha_i+\beta_l)}). \tag{4}$$

The Lagrangian multipliers α_i and β_l are determined from

$$k_i = -\frac{\partial}{\partial \alpha_i} \ln Z = \sum_{l=1}^{M} \frac{e^{-\alpha_i-\beta_l}}{1+e^{-\alpha_i-\beta_l}}, \tag{5}$$

$$d_l = -\frac{\partial}{\partial \beta_l} \ln Z = \sum_{i=1}^{N} \frac{e^{-\alpha_i-\beta_l}}{1+e^{-\alpha_i-\beta_l}}. \tag{6}$$

Finally, we can calculate the probability p_{il} that a member i attended event l. If we define coupling parameter $\lambda_{il} = \alpha_i + \beta_l$ and write partition function in the form

$$Z = \sum_G e^{-\sum_{il} \lambda_{il} B_{il}} = \prod_{il}(1 + e^{-\lambda_{il}}), \tag{7}$$

then, it holds

$$p_{il} = \langle B_{il} \rangle = -\frac{\partial}{\partial \lambda_{il}} \ln Z = \frac{e^{-\lambda_{il}}}{1+e^{-\lambda_{il}}} = \frac{e^{-\alpha_i-\beta_l}}{1+e^{-\alpha_i-\beta_l}}. \tag{8}$$

Now, when the probability p_{il} is given, the probability that members i and j both participated in event l is $p_{ij}(l) = p_{il}p_{jl}$. The probability $P_{ij}(w)$ of having an edge of the weight w between the nodes i and j is given by Poisson binomial distribution

$$P_{ij}(w) = \sum_{M_w} \prod_{l \in M_w} p_{ij}(l) \prod_{\bar{l} \notin M_w} (1 - p_{ij}(\bar{l})), \tag{9}$$

where M_w is the subset of w events that can be chosen from given M events [2,3,6]. We use DFT-CF method (Discrete Fourier Transform of characteristic function), proposed in [7], to compute Poisson binomial distribution.

On the basis of $P_{ij}(w)$, we define p-value as the probability that edge (i,j) has weight higher or equal than w_{ij}

$$p\text{-value}(w_{ij}) = \sum_{w \geq w_{ij}} P_{ij}(w). \tag{10}$$

The edge (i,j) will be considered statistically significant if p-value$(w_{ij}) \leq \alpha$. In our case, threshold $\alpha = 0.05$. If p-value$(w_{ij}) > \alpha$, the edge (i,j) should be removed as spurious statistical connection between members (set $w_{ij} = 0$).

2 Distribution fitting

We fit exponential function $e^{-\lambda x}$, power law function $x^{-\gamma}$ and truncated power law $x^{-\alpha}e^{-Bx}$ to the probability distribution of the total number of participations in group events using the maximum-likelihood fitting method [8]. It is evident from Fig A that the distribution does not follow exponential fit. We compare how the power law and the truncated power law distribution, which are the nested versions of each other, fit the data by calculating the log likelihood ratio $\mathcal{R}$ and π-value (see Ref. [8]). Here, the negative value of $\mathcal{R}$ indicates that the truncated power law is a superior fit to the power law. Additionally, when the value of $\mathcal{R}$ tends to 0, one can use π-value. The small π-value indicates that the power law distribution can be excluded. Table A shows that the truncated power law is a superior fit compared to power law for all four empirical distributions.

3 Data randomization

We randomize event participation patterns preserving the total number of participation for each member and the number of participants per event [9]. Firstly, we choose at random two members, i and j, and for each of them we choose randomly an event they participated, l^i and l^j. If $l^i \neq l^j$, and i didn't participate at event l^j and j didn't participate at event l^i, they are swapped. We perform 10 × (number of participants) × (number of events) swaps. The randomization of the event participation times induces transformations of associated weighted network. The number of participants at some event will stay the same, but participants will differ, resulting in weight increase of certain edges and likewise in weight decrease of some other edges in weighted network. The total weight of the network will be preserved.

For each Meetup group we generate 100 randomized weighted networks and filter out non-significant edges.

4 Figures

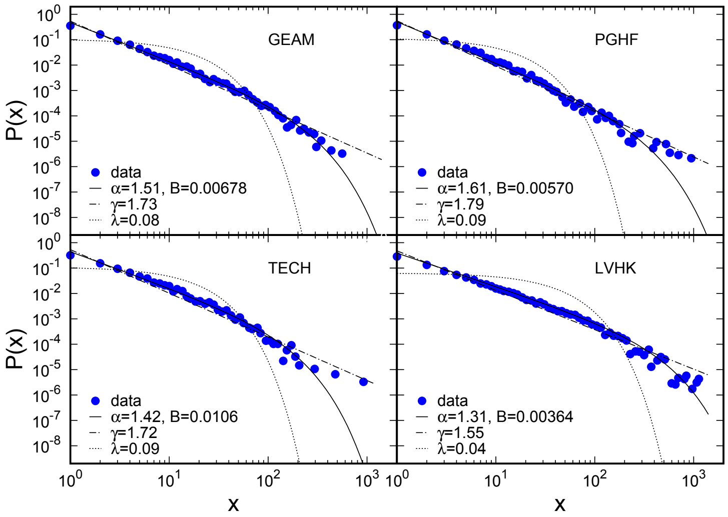

Figure A: The probability distribution $P(x)$ of total numbers of participations in group events x, obtained from the empirical data for the four selected Meetup groups (blue circles). We also show truncated power law fit $x^{-\alpha}e^{-Bx}$ (solid lines), power law fit $x^{-\gamma}$ (dotted-dashed lines), and exponential fit $e^{-\lambda x}$ (dotted lines).

	$\mathcal{R}$	π
GEAM	-97.70	0.0
PGHF	-58.04	0.0
TECH	-90.84	0.0
LVHK	-236.52	0.0

Table A: Log likelihood ratio $\mathcal{R}$ and the π-value compare fits to the power law and fits to the truncated power law for the probability distribution of total numbers of participations in group events.

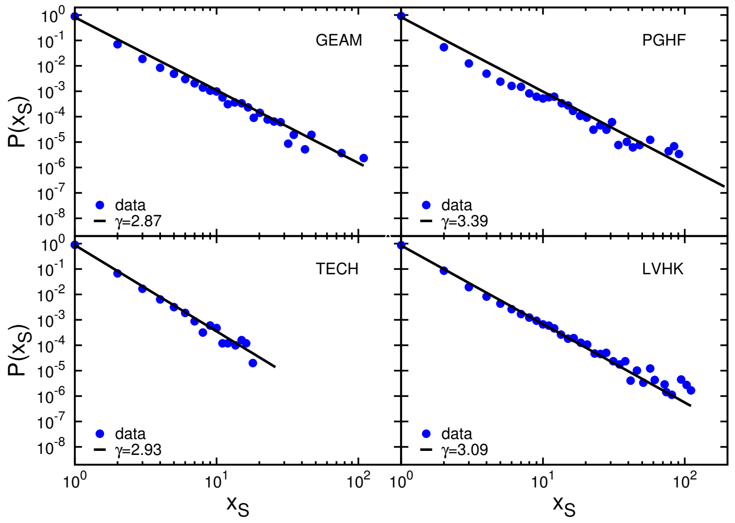

Figure B: The probability distribution of successive numbers of participations in group events x_S, for the four selected Meetup groups. The probability distribution follows power law behavior $P(x_S) \sim x_S^{-\gamma}$.

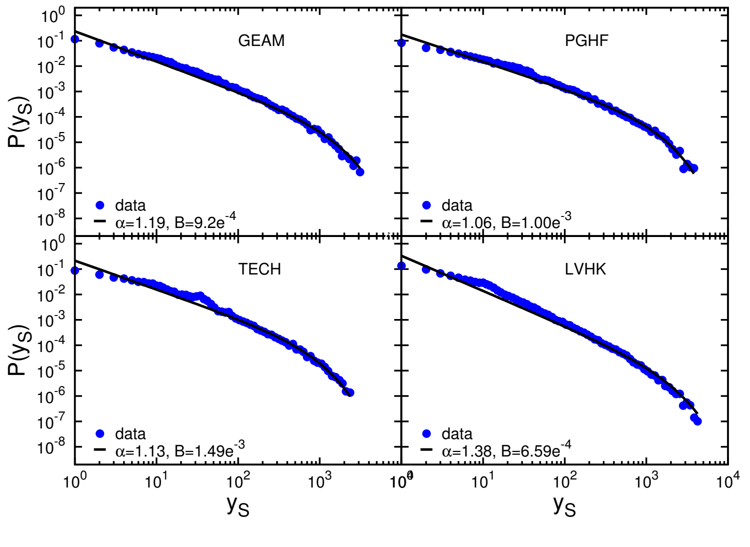

Figure C: The probability distribution of time lags between two successive participations in group events y_S, for the four selected Meetup groups. The probability distribution follows truncated power law behavior $P(y_S) \sim y_S^{-\alpha} e^{-By_S}$.

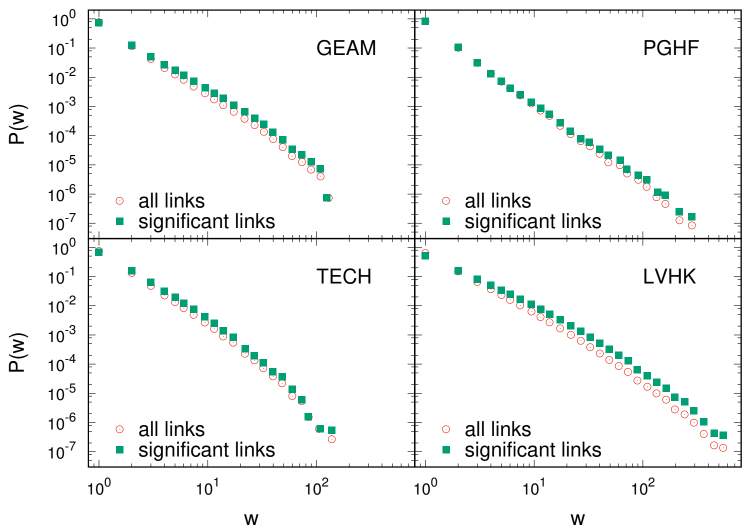

Figure D: The probability distribution of link weights in a weighted network before and after filtering, for the four selected Meetup groups.

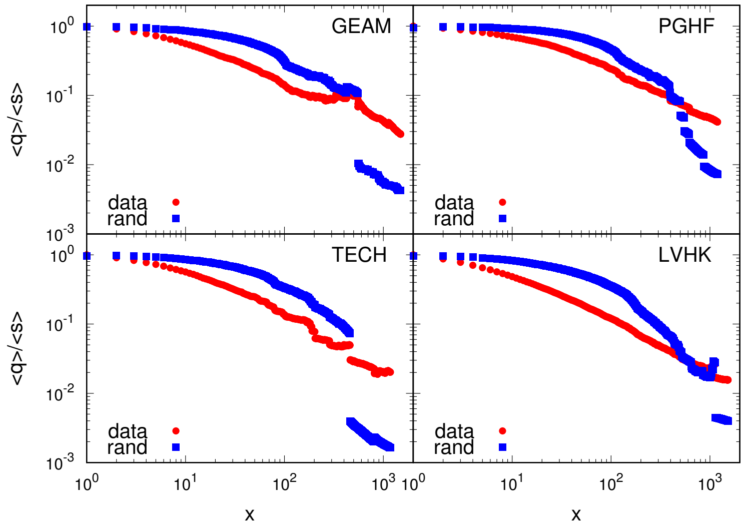

Figure E: The dependence of a degree strength ratio on the number of participations, averaged over all members for the four considered Meetup groups. Red circles correspond to results obtained from empirical data, while blue squares correspond to randomized data.

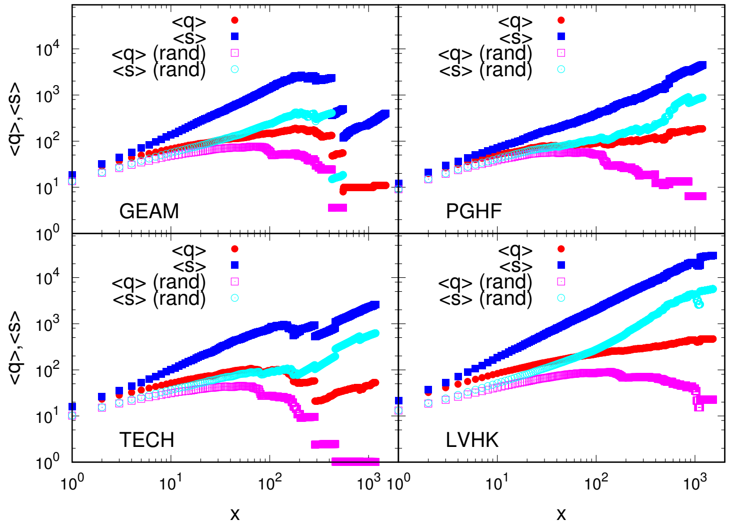

Figure F: The dependence of group members' average degree $\langle q \rangle$ and strength $\langle s \rangle$ on numbers of participations for a real weighted network and a randomized network.

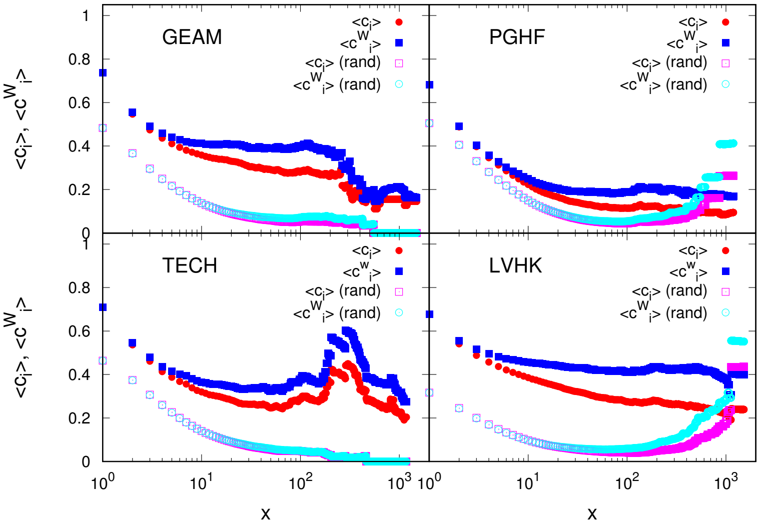

Figure G: The dependence of group members' average non-weighted $\langle c_i \rangle$ and weighted clustering coefficient $\langle c_i^W \rangle$ on numbers of participations for a real weighted network and a randomized network.

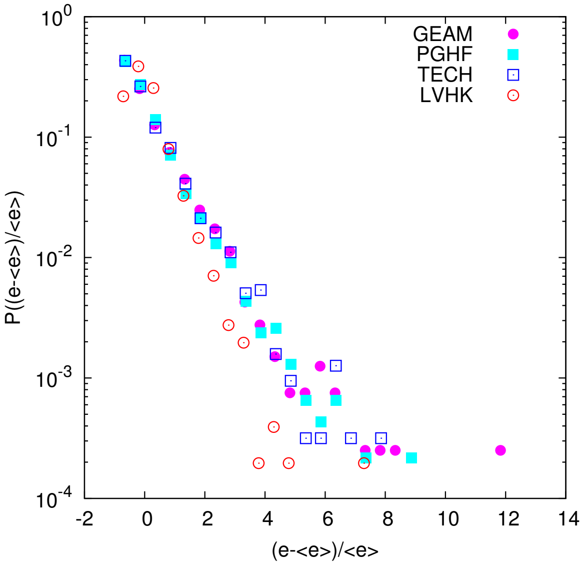

Figure H: The probability distribution of relative size fluctuations $\frac{\langle e\rangle - e}{\langle e\rangle}$, for the four considered Meetup groups, where e is the event size and $\langle e\rangle$ is the average event size.

References

[1] N. Dianati, "Unwinding the hairball graph: Pruning algorithms for weighted complex networks," *Phys. Rev. E*, vol. 93, p. 012304, 2016.

[2] N. Dianati, "A maximum entropy approach to separating noise from signal in bimodal affiliation networks," *ArXiv e-prints*, July 2016.

[3] F. Saracco, R. Di Clemente, A. Gabrielli, and T. Squartini, "Grandcanonical projection of bipartite networks," *ArXiv e-prints*, July 2016.

[4] F. Saracco, R. Di Clemente, A. Gabrielli, and T. Squartini, "Randomizing bipartite networks: the case of the world trade web," *Sci. Rep.*, vol. 5, p. 10595, 2015.

[5] D. Cellai and G. Bianconi, "Multiplex networks with heterogeneous activities of the nodes," *Phys. Rev. E*, vol. 93, p. 032302, 2016.

[6] J. Liebig and A. Rao, "Fast extraction of the backbone of projected bipartite networks to aid community detection," *Europhys. Lett.*, vol. 113, no. 2, p. 28003, 2016.

[7] Y. Hong, "On computing the distribution function for the poisson binomial distribution," *Computational Statistics and Data Analysis*, vol. 59, pp. 41 – 51, 2013.

[8] A. Clauset, C. R. Shalizi, and M. E. J. Newman, "Power-law distributions in empirical data," *SIAM Review*, vol. 51, no. 4, pp. 661–703, 2009.

[9] G. Strona, D. Nappo, F. Boccacci, S. Fattorini, and J. San-Miguel-Ayanz, "A fast and unbiased procedure to randomize ecological binary matrices with fixed row and column totals," *Nature communications*, vol. 5, p. 4114, 2014.
